# Suspended Carbon Nanotubes for Humidity Sensing

**DOI:** 10.3390/s18051655

**Published:** 2018-05-22

**Authors:** Shivaram Arunachalam, Anubha A. Gupta, Ricardo Izquierdo, Frederic Nabki

**Affiliations:** Department of Electrical Engineering, École de Technologie Supérieure, Montreal, QC H3C 1K3, Canada; anubha-a.gupta.1@ens.etsmtl.ca (A.A.G.); ricardo.izquierdo@etsmtl.ca (R.I.); frederic.nabki@etsmtl.ca (F.N.)

**Keywords:** carbon nanotubes, non-suspended, suspended, humidity sensor

## Abstract

A room temperature microfabrication technique using SU8, an epoxy-based highly functional photoresist as a sacrificial layer, is developed to obtain suspended aligned carbon nanotube beams. The humidity-sensing characteristics of aligned suspended single-walled carbon nanotube films are studied. A comparative study between suspended and non-suspended architectures is done by recording the resistance change in the nanotubes under humidity. For the tests, the humidity was varied from 15% to 98% RH. A comparative study between suspended and non-suspended devices shows that the response and recovery times of the suspended devices was found to be almost 3 times shorter than the non-suspended devices. The suspended devices also showed minimal hysteresis even after 10 humidity cycles, and also exhibit enhanced sensitivity. Repeatability tests were performed by subjecting the sensors to continuous humidification cycles. All tests reported here have been performed using pristine non-functionalized nanotubes.

## 1. Introduction

Ever since their discovery in 1991 by Iijima [1] carbon nanotubes have been studied intensively due to their remarkable electrical and mechanical properties. A number of potential applications of carbon nanotubes have been well catalogued in the literature [2,3]. Owing to their excellent electrical properties, the application of nanotubes in the area of sensing has been intensively studied [4]. Enough theoretical and experimental proofs now exist that have led to the notion that carbon nanotubes are one of the future go-to materials of the sensing industry. Over the years, the cost of nanotube manufacturing has significantly decreased, which further makes them an attractive proposition to be used in sensors. Many different types of sensors ranging from pressure sensors [5] to optical sensors [6] have been demonstrated. Other applications where nanotubes are used as sensing materials are solar cells [7], displays [8] and transistors [9]. 

Humidity Sensors play a major role in environment monitoring in various locations such as homes, automobiles and in medicine [10]. A host of materials and methods have been researched upon to improve sensor parameters such as response times, shelf life, selectivity and more importantly to bring down manufacturing costs [11,12,13]. To date, a majority of the work based on carbon nanotube humidity sensors published in the literature have been fabricated using chemically functionalized multiwalled carbon nanotubes [14,15,16]. However, few works have investigated single-walled nanotubes for humidity sensing. For instance, Mudimela et al. [17] fabricated a humidity sensor based on networks of single-walled carbon nanotubes Field Effect Transistors (FETs). More recently, Zhou et al. [18] developed a textile-based humidity sensor based on Poly vinyl alcohol (PVA) functionalized single-walled nanotubes with a response time of 40 s. Carboxylic acid functionalized single-walled nanotube networks were used on a cellulose paper template to sense humidity by Han et al. [19] with a fast response time of about 6 s. All the above-mentioned works have used either single or a network of substrate bound carbon nanotubes. The one common problem that all the devices experience is hysteresis which can lead to reduced reliability. One way to eliminate the problem of hysteresis is to use suspended carbon nanotubes as the sensing material. Suspended carbon nanotubes were used to detect NO_2_ with minimal hysteresis [20]. However, suspended nanotubes are difficult to fabricate, leading to only a few suspended nanotube sensors in the literature. Suspended aligned nanotube networks were fabricated by Lee et al. [21] using a microfluidic template. Well aligned nanotube networks have shown to have better electrical and mechanical properties [22,23]. More recently, suspended carbon nanotubes were obtained using a transfer approach [24]. However, it is interesting to note that there has very little work done to investigate how aligned suspended carbon nanotube networks would react to humidity. Having a suspended architecture can be very useful in sensing applications due to the increased surface area for adsorption. In this work, we have used SU8, an epoxy-based negative photoresist as a sacrificial material in a low-temperature surface micromachining process to obtain suspended beams comprising of networks of carbon nanotubes. The low-temperature of the process makes it notably suitable for the integration of the suspended beam directly above CMOS integrated circuits. The suspended nanotubes beams were then tested to quantify their sensitivity to humidity. We compare the performance of the suspended nanotubes beam humidity sensor to a non-suspended nanotube beam sensor. Our results indicate that the response and recovery times of a suspended architecture are almost three times lower than a non-suspended architecture without any chemical modification to the nanotubes.

## 2. Materials and Methods

**Figure 1:**
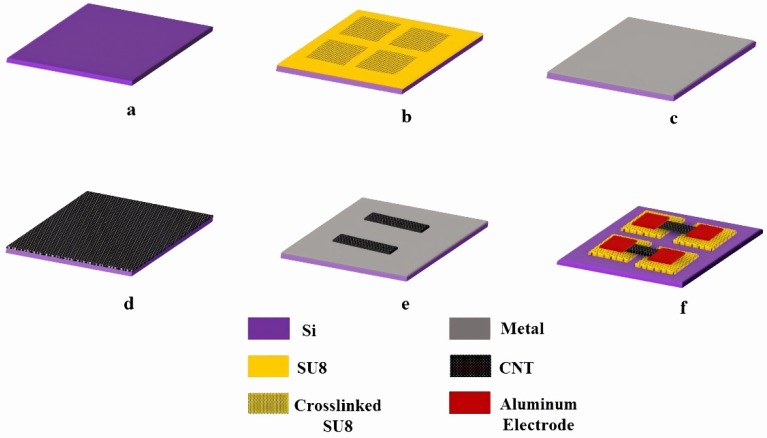
Process flow diagram.

Figure 1 shows the schematic of the fabrication process. The single-walled carbon nanotubes in this work were obtained from Carbon Solutions Inc and used without any further modifications. The as obtained nanotubes were dispersed in a 1% Sodium Dodecyl Sulfate (SDS) solution and sonicated for 4 h. After sonication, the solution is centrifuged for 60 min to remove any solid nanotube agglomerates. The top 80% of the centrifuged solution is decanted for further use. 

The process starts with a Silicon wafer with a 300 nm oxide (Figure 1a). A 3.5 µm thick SU8 layer is spun and then it is selectively UV exposed using an OAI Hybralign system (Figure 1b). The exposed SU8 crosslinks and acts as “pillars” for the nanotube beams. A 100 nm thick Aluminum layer is evaporated on top of the SU8 layer using filament evaporation, without developing the uncrosslinked SU8 (Figure 1c). This metal layer acts as a barrier and protects the un-crosslinked SU8 from being attacked by organic solvents in future processing steps. The temperature inside the evaporation chamber is verified using temperature strips obtained from Thermax in order to ensure that the SU8 is not cross-linked during the evaporation process. The next step is to deposit the nanotube film (Figure 1d). The carbon nanotube film was created using a simple vacuum filtration method [25,26]. 

Vacuum filtration enables the formation of a uniform nanotube network and allows a precise control over the thickness of the film depending on the amount of solution used. More importantly, vacuum filtration is a room temperature deposition method and is of low-cost compared to other techniques. The nanotube film is deposited on a nitrocellulose membrane. After vacuum filtration, the film is washed in deionized (DI) water to remove any traces of surfactant that may be present on the surface. Then, the carbon nanotube film is transferred onto the substrate. The film is cut into the desired shape and size and transferred onto the substrate by dipping the nanotube film in chlorobenzene. The substrate is then dipped in acetone for 60 min to dissolve the nitrocellulose membrane. The resulting nanotube film thickness is between 0.5–0.7 µm depending on the amount of solution used. The nanotube film is then patterned by UV lithography. After development, the nanotube film is etched using oxygen plasma (Figure 1e). After the plasma step, the barrier metal is etched by wet etching. The next step of the process is to remove the uncrosslinked SU8 by dipping the substrate in Developer for 60 s to release the suspended structures, and the final step of the process is to deposit 50 nm thick Aluminum electrodes (Figure 1f). For the non-suspended carbon nanotubes, the nanotube film was directly transferred onto the substrate, patterned and etched using oxygen plasma using the same gas flow rates and chamber pressures as in the case of suspended carbon nanotube devices to minimize variability. Aluminum electrodes were then patterned onto the nanotubes for electrical measurements. 

The concept of using SU8 as a sacrificial material was adopted from [27]. The reason for selecting SU8 as a sacrificial layer is the ease of availability and the thermal budget it provides. It also provides a very stable surface for further chemical processes and is also compatible with surface micromachining. Many polymeric materials as a sacrificial material were also considered, but it was found that using these materials lead to cracking of the metal barrier layer with the metal layer subsequently peeling off. This could be due to the thermal stress developed at the interface of the metal-polymer layer, which reduces the adhesiveness of the metal to the sacrificial layer. The other reason is the ease of removal of the SU8. Uncrosslinked SU8 can also be very selectively removed in the presence of other materials as mentioned in the referred work. Importantly, the process proposed in this work is low temperature and can be incorporated above CMOS integrated circuits. 

## 3. Results and Discussion

The samples were imaged using a MEB 3600 Scanning Electron Microscope. The samples were metallized by sputtering a 20 nm Gold layer to make the nanotubes conductive for the SEM. It was found that the sputtering did not affect the properties of the nanotubes. 

**Figure 2:**
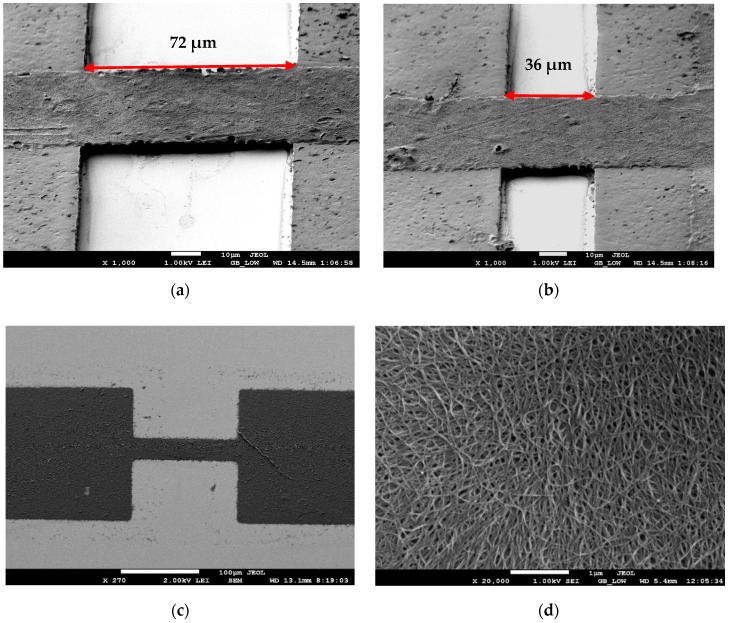
(**a**) and (**b**) suspended beams with different suspension lengths, (**c**) a normal non-suspended nanotube beam, and (**d**) networks of nanotubes that comprise the beam.

Figure 2a,b shows Scanning Electron Microscope (SEM) micrographs of suspended nanotube beams across the SU8 “pillars”. Figure 2c shows a conventional non-suspended device, and Figure 2d shows a high magnification micrograph which shows the networks of nanotubes comprising the beam. The nanotube beam is suspended at a height of 3.5 µm above the substrate. After fabrication, the humidity-sensing characteristics of both types of sensors are recorded by measuring the change in resistance of the devices.

**Figure 3:**
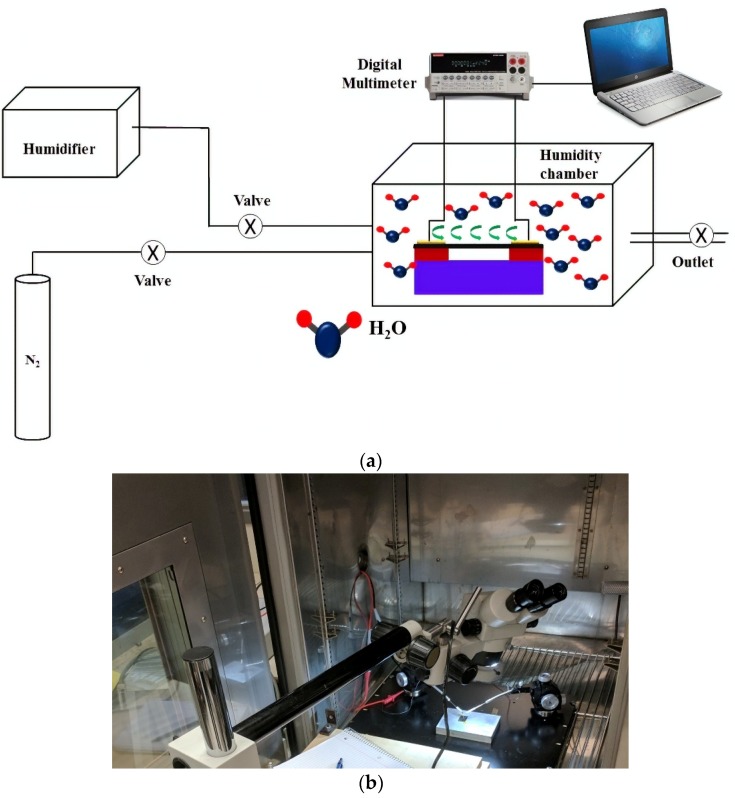
(**a**) Schematic of the test setup, and (**b**) Photograph of the device inside the test chamber.

Figure 3a,b shows the schematic of the test setup and a photograph of the setup used for the measurements. The sensors were interconnected using probes aligned with micro positioners. A humidity chamber with an external humidity source was used for the measurements. The initial humidity was of 15% RH. The percentage humidity inside the chamber was verified using a high-precision sensor embedded within the humidity chamber. The compressor inside the humidity chamber could not be used to produce humidity since the vibrations led to the probes scratching the metal electrodes and destroying the devices. Accordingly, an external humidifier was used to inject water vapor into the chamber and a nitrogen tank was used to quickly flush out the humidity when required. Nitrogen was chosen as a carrier gas in order to maintain an inert environment within the chamber. The resistance of the devices was measured using a Keithley Digital Multimeter. The multimeter was in-turn connected to a computer for data acquisition. The resistance was allowed to stabilize inside the chamber for 30 min prior to the first measurement.

**Figure 4:**
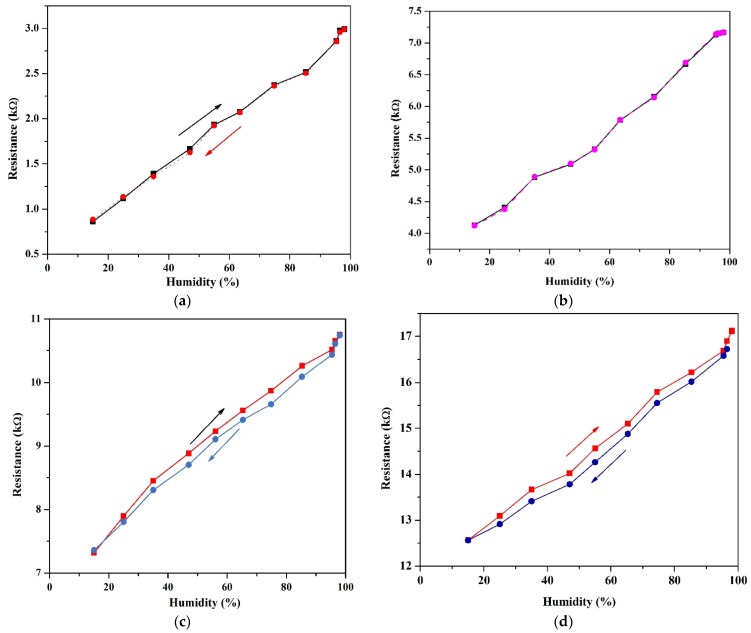
(**a**,**b**) humidity response and hysteresis for suspended carbon nanotubes (36 µm and 72 µm respectively), and (**c**,**d**) humidity response and hysteresis for non-suspended carbon nanotubes (36 µm and 72 µm respectively).

Figure 4 shows the response of both suspended and non-suspended devices for relative humidity ranging from 15% to 98% RH. Figure 4a,b shows the hysteresis characteristics and average resistance values of suspended devices with different suspension lengths averaged for 10 up-and-down cycles respectively. The base resistance was around 0.86 kΩ for the 36 µm suspended nanotubes (Device A) and 4.12 kΩ for a device with 72 µm suspension length (Device B) at 15% humidity. At 98% humidity, the resistances for devices A and B are 2.991 kΩ and 7.167 kΩ, respectively. The suspended devices showed minimal hysteresis even after multiple humidity cycles. 

As shown in Figure 4c,d, for the non-suspended devices, devices with similar channel lengths of 36 µm (Device C) and 72 µm (Device D) had a base resistance of 7.3 kΩ and 12.56 kΩ respectively at 15% humidity. At 98% humidity, the resistance was 10.755 kΩ and 17.11 kΩ for devices C and D, respectively. The devices showed an almost linear response to humidity. The non-suspended devices exhibit similar hysteresis characteristics. The hysteresis is caused by water molecules on the substrate, agreeing with the work reported in [28]. The high resistance of the non-suspended devices is possibly due to the presence of charge traps and defects within the substrate which limit the flow of charge carriers in the network while in the case of suspended devices, the lack of substrate interactions provides a smoother pathway for charge carriers.

The humidity-sensing mechanism of the nanotubes has been extensively studied and described in works such as [29,30]. The nanotubes are inherently p-type having holes as the majority charge carriers. When a water molecule interacts with the nanotube, it donates electrons to the nanotubes and because of electron transfer, the number of effective charge carriers in the nanotube decrease thereby increasing the resistance of the devices.

**Figure 5:**
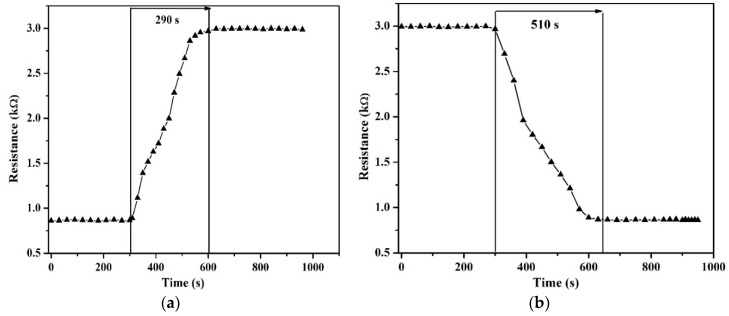
(**a**) Rise time and (**b**) fall times of suspended carbon nanotubes. The humidity was varied from 15% to 98% in steps of 10% RH increases every 20 s.

**Figure 6:**
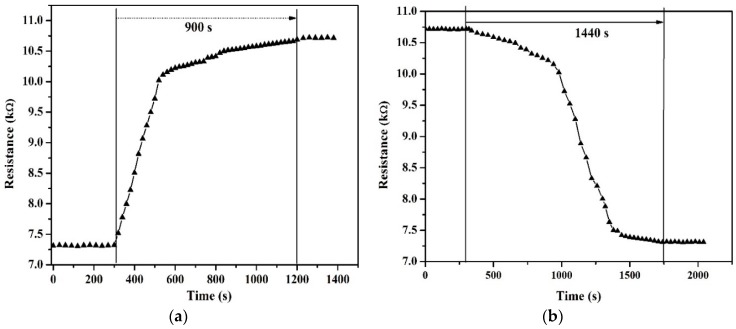
(**a**) Rise time and (**b**) fall time of non-suspended carbon nanotubes. The humidity was varied from 98% to 15% RH in steps of 10% RH decreases every 20 s.

Figure 5a,b and Figure 6a,b show the rise and fall times of both types of sensors. The response time of the suspended structures is 290 s as compared to 900 s for the non-suspended devices. The faster rising response of suspended structure is due to the increased surface area of the device thanks to the flow of humidity below the beam enabled by the suspension of the nanotubes beam. The recovery or fall times are 510 s and 1440 s for suspended and non-suspended devices, respectively, outlining again the advantage of the suspended structure. 

**Figure 7:**
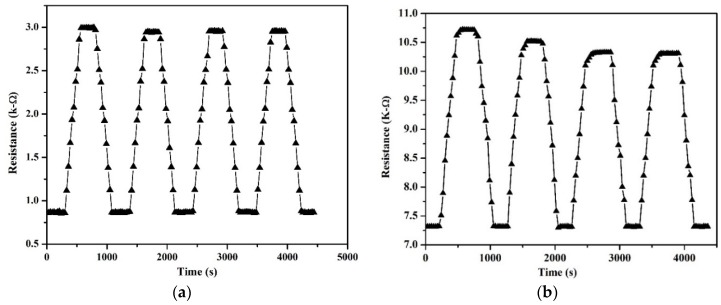
Repeatability of (**a**) suspended carbon nanotubes, and (**b**) non-suspended carbon nanotubes.

The longer recovery time of the non-suspended devices could be due to the presence of water molecules on the nanotube network during the desorption process and to the reduced surface area available for desorption. Figure 7a,b shows repeatability of the devices by plotting resistance as a function of time for four continuous humidity cycles under continuous humidification from 15% to 98% RH. The resistance of each device was allowed to stabilize for a few minutes before the beginning of each cycle. The suspended humidity sensor showed very consistent resistance values even after 3 cycles whereas the non-suspended sensor began to show drifting performance, outlining the compromised dynamics due to the reduced surface area and absent gas flow below the beam. 

**Figure 8:**
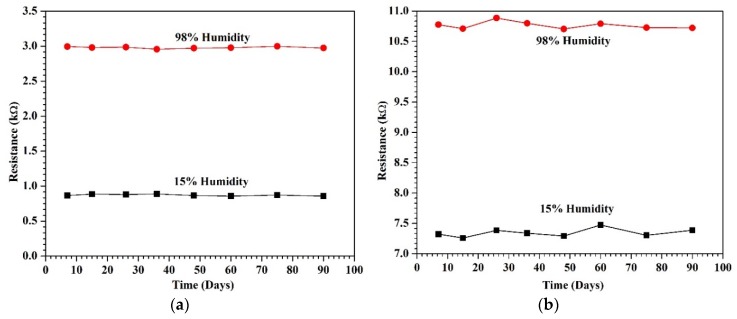
Long-term stability of (**a**) suspended sensors and (**b**) non-suspended sensors of 36 µm.

One of the most important parameters for gauging sensor performance is long-term stability. The sensor should show consistent values and response even after prolonged duration. To demonstrate long-term stability, the resistance of the suspended devices was measured after certain time intervals with similar testing conditions as the initial tests. The suspended nanotube sensors exhibited stable performance over the time period as shown in Figure 8. 

**Figure 9:**
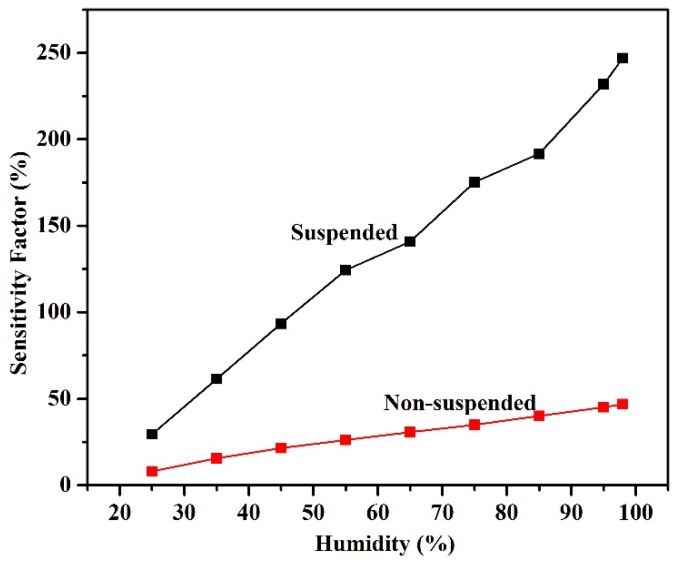
Sensitivities of suspended and non-suspended sensors.

It has been shown in this work that the sensitivity of the sensor is improved by the suspension of the carbon nanotubes beam. The sensitivity factor, in percent, is given by

(1) $$S=\frac{R_{H}-R_{0}}{R_{0}}\times 100$$

where *R_H_* is the resistance at the measured value of resistance, and *R*_0_ is value of baseline resistance. Since the suspended nanotubes beam is not connected to the substrate, the water molecules have both the top and bottom surface to adhere to which increases the sensitivity of the sensor. Figure 9 shows a plot of the sensitivity factor of both suspended and non-suspended sensors as a function of the relative humidity. 

It can be gauged from this plot that the sensitivity of the suspended nanotubes is much greater than the non-suspended networks. From a base humidity of 15%, the increase in resistance at 98% humidity was of 246.9% for the suspended device compared to only 46.83% for the non-suspended devices. The results of this study are a clear indication that the use of suspended structures for sensing applications yields significant advantages in comparison to traditional substrate bound materials. It is clear that the response and recovery times presented in this work are not suitable for commercial use, as the nanotubes have not been functionalized. However, the aim of this work is to demonstrate the advantages of suspended nanotube networks, in terms of response time, hysteresis and sensitivity. Many functionalization schemes have been reported in the literature [19,31,32] and could be used to improve response time, while benefiting from the suspended structure. The recovery time can also be further improved by incorporating a micro-heater into the device to promote heating and therefore faster desorption. The comparison that is provided here does nonetheless outline the advantages of suspending the carbon nanotubes beam in order to improve response time, sensitivity and hysteresis.

## 4. Conclusions

Suspended carbon nanotubes have been investigated as an alternative to conventional non-suspended devices. We have demonstrated an easy, low-temperature fabrication process to obtain suspended carbon nanotube beams using a common polymer sacrificial layer. Notably, the low-temperature of the process is well suited to the integrated of devices directly above CMOS integrated circuits. Moreover, this process with some modifications can also be used to create devices such as nanotube micromechanical resonators. 

It was found that suspended carbon nanotubes have better all-round humidity-sensing performance as compared to non-suspended architecture. The suspended nanotube sensor exhibited repeatable performance with good response times and recovery times as compared to the non-suspended sensor. The significantly reduced hysteresis in the suspended nanotube humidity sensor is a major advantage, along with their enhanced sensitivity and response time. The performance of the devices can be enhanced by chemical modification of the nanotube network and future work is to functionalize the nanotubes and study their humidity response in a suspended architecture.
